# Anatomical Notes

**Published:** 1880-04

**Authors:** 


### ANATOMICAL NOTES.

---

3. REMARKABLE GLANDS ON THE SIDE OF THE DOG'S AORTA.—Young dogs possess either singly on one, or doubly on both sides of the assending aorta, brownish glandular substances of the same structure as the thyroid gland, and as large as a lentil. They were found muchex ceeding these dimensions in a dog afflicted with goitre. They have been found four times out of ten in the new-born human being. From their structure, and their evident sympathy (pathologically established) with the thyroid gland, they merit the designation of aortic thyroid glands.—*Centralblatt fuer Chirurgie, 1879. No. 36.*

---

4. A LINE OF DEMARCATION BETWEEN THE SAUROPSIDA AND MAMMALIA ABOLISHED.—The *dictum* that the Mammalia alone possess Corpora Quadrigemina, and that their *four* tubercles are represented by only two bodies in reptiles (Huxley, Claus, Gegenbaur), is erroneous. The posterior pair was found, as a concealed lentiform ganglion in the region intervening between the optic lobes (corpora bigemina) and the cerebellum, in turtles and other ordinary reptiles examined. A distinct elevation visible on the surface is noticeable in the sheltopusik (*pseudopus*) and anaconda (*eunectes*), but in no reptile so beautifully marked as in the iguana, where, though smaller, they are as distinct as in any mammal. It is to defective dissection that the oversight of this important relation, which has led to the erroneous inference mentioned, is due.—*N. Y. Medical Record, March.*

---

5. THE BRAIN OF THE IGUANA.—A more extended study of this brain, preliminary to a thorough microscopical analysis, demonstrates the following noteworthy features: The anterior pair of the corpora quadrigemina is very large, and at the basilar surface there seems to be a distinct corpus geniculatum (externum) in the shape of a tubercular elevation of the optic tract. The posterior pair is covered by the antiflected cerebellum, which, on its ventricular face, is raised into two symmetrical prominences by the posterior pair. The trochlearis, or fourth pair, is given off *behind* this posterior pair. A distinct nerve tract appears to enter the anterior pair from the medulla, by the side of the posterior pair; this is perhaps analagous to the lemniscus superior of mammals. The pedicles of the olfactory bulb are very thin, as in the alligator. The immense size of the optic lobes makes the hemispheres appear proportionately small, though, in reality, they exceed those of the alligator, relatively. No other animal, not excluding birds, has such large optic lobes to my knowledge.

E. C. SPITKZA, M. D.

---

6. THE BRAIN OF THE PTEROPUS.—I have obtained the brains from five specimens of the large fruit-bat, or "flying fox" of New Zealand. Two features are remarkable, and deserve mention. First, the anterior pyramids, on emerging from the Pons Varolii, after running about one millimeter free and distinct, side by side, *decussate superficially*, and the decussated tract can be distinctly traced on the surface of the lateral columns for a distance of from two to seven centimeters. In one brain, the tract of the right side crosses *bodily* over that of the left; in others, the fasciculi cross alternately; in still others, there is a confused mass of fibres from which the crossed part emerges as distinctly as in the other specimens. Microscopical investigation supports the inference that it is a true crossing, and complete. I have thought that the well-marked individuality of this crossing was related in some way to the preponderating importance of the anterior extremities. The second feature is the disproportion between the anterior and posterior pairs of the corpora quadrigemina. The anterior pair is at least five times as bulky as the posterior, reversing the condition in the crepuscular bats, whose anterior pair is smaller than the posterior. The fornix, as in other small mammals, can be readily traced into the thalamus directly, and it appears to me that this mode of termination suggests the homology of the human taenia semicircularis or stria cornea. E. C. SPITZKA, M. D.

---

7. Embryonic Asymmetry.—In a human embryo of about twenty-three days, received from Dr. Ferdinand, I note that the anterior extremity of the right side is twice as long and voluminous as that of the left. There is a distinct tail, which is curved to the left side in half a spiral. In an embryo aged four weeks, the symmetry of the anterior extremities is almost entirely re-established. The inferior extremities are at all times symmetrical. It was also observed that the fourth branchial (visceral) arch disappears earlier on the right than on the left side.

E. C. SPITZKA, M. D.

---

